# Early detection of rumors based on source tweet-word graph attention networks

**DOI:** 10.1371/journal.pone.0271224

**Published:** 2022-07-11

**Authors:** Hao Jia, Honglei Wang, Xiaoping Zhang

**Affiliations:** 1 School of Electrical Engineering at Guizhou University, Guizhou University, Guiyang, Guizhou province, China; 2 The Key Laboratory of “Internet +" Collaborative Intelligent Manufacturing in Guizhou Province, Guiyang, Guizhou province, China; 3 The Science and Technology Department of Guizhou Province, Guiyang, Guizhou province, China; Hanyang University, REPUBLIC OF KOREA

## Abstract

The massively and rapidly spreading disinformation on social network platforms poses a serious threat to public safety and social governance. Therefore, early and accurate detection of rumors in social networks is of vital importance before they spread on a large scale. Considering the small-world property of social networks, the source tweet-word graph is decomposed from the global graph of rumors, and a rumor detection method based on graph attention network of source tweet-word graph is proposed to fully learn the structure of rumor propagation and the deep representation of text contents. Specifically, the proposed model can adequately capture the contextual semantic association representation of source tweets during the propagation and extract semantic features. For the data sparseness of the early stage of information dissemination, text attention mechanism based on opinion similarity can aggregate and capture more tweet propagation structure features to help improve the efficiency of early detection of rumors. Through the analysis of the experimental results on real public datasets, the rumor detection performance of the proposed method is better than that of other baseline methods. Especially in the early rumor detection tasks, the proposed method can detect rumors with an accuracy of nearly 90% in the early stage of information dissemination. And it still has good robustness with noise interference.

## 1. Introduction

The vigorous development and iteration of network technology and electronic devices have made social networks an indispensable part of people’s daily lives. The emergence of social network platforms such as Twitter, Yelp, and Reddit has greatly facilitated people to quickly obtain and exchange information through virtual networks. By the end of 2020, the number of registered users of Twitter has exceeded 1 billion, the average number of monthly active users had exceeded 330 million, and the total number of tweets sent daily exceeded 500 million. Twitter has become the second-largest social media platform in the world. As one of the most popular social networking platforms, Twitter gives people a lot of information every day and is considered an important news source, which means that information usually spreads faster than traditional media [1]. These social networking platforms have greatly facilitated people to freely create and share information, but they are often filled with a large number of fake news and rumors. The explosive spread of false information poses a threat to the credibility of legitimate online platforms and resources, and has serious negative impacts on individuals and society [2], with the potential consequences of destabilizing society and affecting fair competition [3]. For example, During the global fight against the COVID-19 flu, rumors have flooded the Internet, people may believe that eggs are contaminated with the coronavirus, or that bleach can kill the virus, among other things. These rumors or false information will not only cause negative emotions in the public but may also harm people’s efforts in the epidemic. Fake news and rumors about the new coronavirus have killed hundreds of people, according to a study in 87 countries.

Therefore, it is very necessary and beneficial for society to detect the large amount of false information spread on social media as early as possible, which can prevent rumors from harming public safety and misleading citizens. It is necessary to develop an effective method that can identify different types of rumors with higher accuracy in the early stage of information dissemination. Furthermore, misclassifying and blocking the spread of fake news or information can be counterproductive, such an inappropriate action will affect the freedom and fairness of information sharing on social platforms [4].

Most of the current work mainly considers the text content features, user features, and retweet propagation features of rumors, and extracts such features to realize rumor detection. However, these methods are often limited in extracting features, mainly due to the following reasons. Firstly, the dissemination of information in social networks often takes the form of short texts, and it is difficult to achieve accurate rumor detection with text content features extracted from a single short text [5]. Secondly, linguistic features in rumors are often deceptive to evade existing rumor detection models. Some part of studies attempts to extract other features in information dissemination, including user node information and network structure to detect rumors [6]. However, in practice, obtaining user profiles usually requires consideration of unavoidable issues such as protecting user privacy. At the same time, another part of the studies considers adding structural features to rumor detection. [4, 6–8] use graph convolutional neural networks (GCN) and their variants to build a global propagation graph, combining textual information or user profiles in rumors for rumor detection. Although the method considering structural feature learning has achieved good results to a certain extent, the tweets propagated in the early stage of social media platforms usually have a small amount of data and the network propagation structure is sparse. Therefore, how to fully extract the features of the text contents and combine the features of the propagation structure to realize the early rumor detection task still deserves further research.

To solve the above problems, in the current paper, rumors are considered a claim that may or may not be true at the time that it is posted on Twitter. Rumor detection on Twitter specifies whether the sets of incoming tweets are rumors or not [8]. Numerical results of existing studies confirm that real-world networks, including Twitter, tend to be large-scale small-world networks with high clustering coefficients and short link paths. This kind of network neither conforms to the characteristics of geometric regular graphs nor the characteristics of random graphs and is called a complex network [9]. Therefore, this paper believes that the problem of rumor spreading on social networks is closely related to the publisher, the communicator, and the friends the communicator contacts, and the network topology is the small-world network. All textual information in the process of information dissemination has potential contextual semantic association features, and these features play an important role in improving the early detection accuracy of rumors.

In this study, a method for rumor early detection based on graph attention network is proposed to learn the contextual semantic association representation of source tweets and named STWA, which can jointly learn the source tweet contextual semantic association representation of rumors as well as the source tweet propagation structure features. This study evaluates the performance of the proposed method STWA on the rumor datasets. Through the analysis of the experimental results, the rumor detection performance of the proposed method is better than that of the baselines, especially the performance on the task of early detection of rumors is better than the existing methods. The academic contributions of this study are as follows:

- In this paper, Considering the small-world property of social networks, this research decomposes the source tweet-word graph based on the global graph in the data processing work. The model can capture the propagation structure features and contextual semantic association representations of source tweets more effectively in this decomposition graph, which contributes to feature extraction efficiency and achieves higher rumor detection accuracy.
- This study proposes a text aggregation attention mechanism based on opinion similarity. Adding the calculation of edge connection weights based on opinion similarity in the model can make the model further learn the structure of the propagation graph and obtain more propagation structure features. Therefore, it can resist more influence of the interference of noise in the early detection task and achieve more efficient rumor detection in the early stage of information dissemination.

The present paper is organized as follows: Related works are reviewed in Section 2. A problem statement and detailed explanation of the main aspects of the proposed method STWA are presented in Section 3. A quantitative evaluation of the proposed model is carried out in Section 4. Section 5 concludes and briefly analyzes the direction of future work.

## 2. Related works

The present paper proposes a rumor detection method based on graph attention neural network. The current related work in this field is mainly based on traditional machine learning and deep learning for feature extraction. These features include content-based, user-based, and propagation-based features to complete the classification tasks in rumor detection and verification.

### 2.1 Approaches based on traditional machine learning

In current, most of the methods for early rumor detection are based on traditional machine learning, which considers starting from the text content features, user features, and communication structure features in the dataset, and extracting such features to realize rumor detection. Combining different types of features, Castillo et al. [10] made great contributions to the feature engineering detection task, they proposed detection methods for different types of features, including rumor detection based on text, user, topic, and propagation structure. At the same time, Kwon et al. [11] considered the influence of temporal changes and modeled time series to detect rumors, and their experiments proved that temporal features are useful for rumor detection.

For the task of early rumor detection, the above two works try to use statistical text features to capture the features of text contents of source tweets or retweets to achieve early detection of rumors. Further, to obtain the structural features of rumor propagation. Ma et al. [12] proposed a method based on the time-series features of the rumor life cycle to capture the contextual features of tweets. Wu et al. [13] exploited topological features extracted from the spread of rumors’ source tweets to identify fake information. In research on the structural features of context propagation, Vosoughi et al. [14] established a human-machine collaborative system for rumor detection, which works by collecting features from original tweets at a certain time and inputting them into the system, tweets with similar features will be extracted for detection. Qazvinian et al. [15] used a system to detect rumors that have been discovered. Experimental results on five topics with different dialogue structures show that the method has higher detection accuracy on rumor datasets with longer lifetimes.

However, methods to manually extract features are time-consuming and labor-intensive, and these features are dataset-dependent and sometimes impossible to extract. Therefore, some deep learning models that can automatically extract rumor features are proposed.

### 2.2 Approaches based on deep learning

In recent years, deep learning has achieved some success in many fields, such as artificial intelligence including natural language processing (NLP). More scholars have begun to pay attention to the application of deep learning in rumor detection tasks. Many research results have demonstrated that the ability of these methods to extract language features is significantly enhanced, which can improve the performance of the model [4].

Ajao et al. [16] provided a fusion model based on Convolutional Neural Network (CNN) and Long short-term memory (LSTM) for fake news detection. Chen et al. [17] proposed an RNN-based deep attention model to learn temporal hidden representations of sequential tweets and identify distinct features by learning latent representations from consecutive tweets. Asghar et al. [18] proposed a model fused with bidirectional long short term memory (BiLSTM) and CNN, using BiLSTM to obtain contextual connections in tweets with contextual information, and using CNN to extract tweet features for identifying rumors.

To complete the task of early rumor detection, some scholars have considered using deep learning models to automatically extract relevant features from source tweets. Ma et al. [5] proposed a Recurrent Neural Network (RNN) with Gated Recurrent Unit (GRU) to model the sequential structure of related tweets to capture the temporal information of source tweet propagation. After that, Ma et al. [19] put forward a propagation tree-based RNN model and learned topological features of source tweets to capture propagation and semantic information for rumor detection. Xu et al. [20] proposed a combined neural rumor detection model, which uses an attention mechanism to capture keywords in source tweets and important retweeted content. It aimed to detect rumors through the source tweet contents, retweet contents, and user profiles. Liu et al. [21] attempts to extract user features in source tweets and proposed a classifier learning combining RNN and CNN to propagate the structure to complete the task of rumor detection. Ruchansky et al. [22] developed a framework to capture text, user, and dissemination of structural information for more rumor features. Huang et al. [23] constructed a user graph based on user behavior using a graph convolutional network (GCN), and obtained user representations from the graph combined with a propagation tree for rumor detection.

Recent studies have demonstrated the high efficiency of using deep learning models on graph structures to solve problems in NLP [24]. Compared with other methods, Graph Neural Network (GCN) can capture the overall structural features of the propagation graph [8]. Bian et al. [25] tried to obtain the discontinuous global structure in rumors and proposed a GCN-based deep learning model. Dong et al. [26] built a GCN-based rumor source discrimination model, which still had a good detection performance without the input of basic propagation model knowledge. Tu et al. [6] proposed a method named Rumor2vec, which can merge the joint graph of all tweet propagation structures to alleviate the problem of information sparsity and conduct rumor detection through joint text and propagation structure representation learning. Chen et al. [4] put forward a method based on propagation graph structure and fine-grained user representation learning to learn more explicit and implicit features of user profiles, named PLRD. Lu et al. [27] proposed a co-attention network-based method to detect rumors by fusing source tweet content with users’ information. To solve the problem of Chinese rumors on Weibo, Bi et al. [28] developed a method to achieve efficient rumor detection by combining the features of node graph and semantic graph, which has achieved good results in the improvement of detection accuracy. Although scholars have achieved certain results in the problem of rumor detection, the current methods based on user profiles have to consider the protection of user privacy and the difficulty of data acquisition. Moreover, how to achieve high rumor detection accuracy in the early stage of information dissemination still needs further research.

Few methods based on text and propagation structure take into account the learning of semantic association representations between source tweets and retweets in combination with the propagation graph topology. What’s more, in the early stage of information dissemination, the problem of network data sparseness is still a major challenge for early rumor detection. This paper argues that the implicit features of rumor text content are not effectively extracted, especially the contextual semantic association features of source tweets, which may help improve the early detection accuracy of rumors.

**Fig 1:**
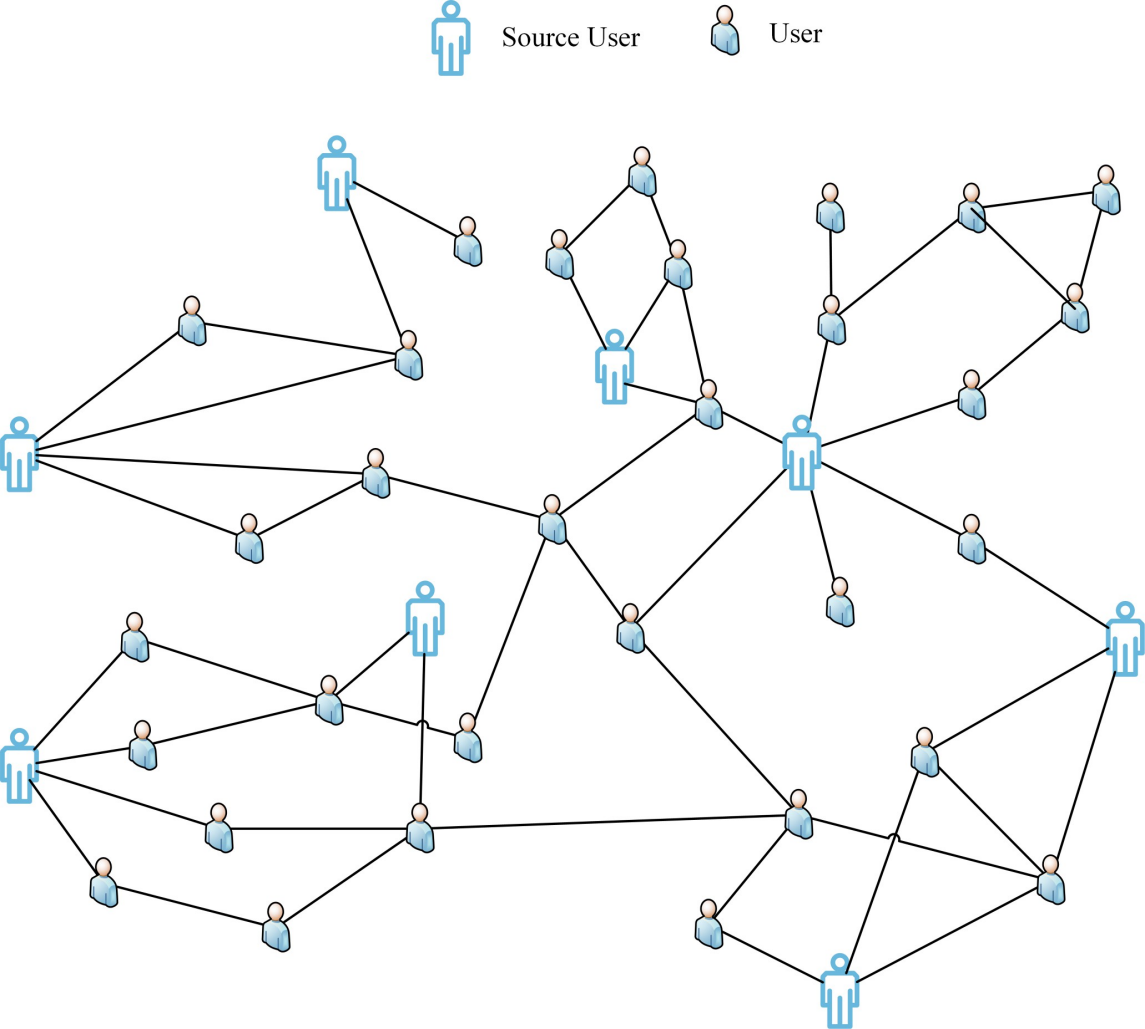
Twitter global graph of network topology.

Therefore, in this study, a graph attention network-based method is used to model the Twitter information dissemination structure. the proposing method in this paper aims to establish a rumor detection method to capture the text content features and propagation structure features of source tweets as many as possible and achieve high detection accuracy in the early stage of rumor propagation. What’s more, with the increase in noisy data, the model still has good robustness in the case of sparse data. Specifically, Considering the small-world properties of social networks, a global graph of the tweet propagation process is built in this paper, as shown in Fig 1.

## 3. The proposed method

In this section, we first illustrate how to construct the Twitter global graph and the source tweet-word graph, and make a preliminary statement on the rumor detection problem. After that, the overall framework of the proposed method STWA and the details of two modules of the source tweet-word graph attention network and the text attention mechanism based on opinion similarity included in the framework are described in detail. In this study. The two main challenges that need to be addressed to develop an effective rumor early detection model in this study are as follows: (1) How to capture the semantic association representation between a particular source tweet and the retweet text during the propagation process. (2) In the case of sparse data in the early stage of Twitter network propagation, how to ensure that the model learns the explicit and implicit representations of all Twitter text content features as fully as possible. For solving the above two problems, a global graph that meets the requirements of this paper is first constructed.

### 3.1 Construction of source tweet-word graph

**Fig 2:**
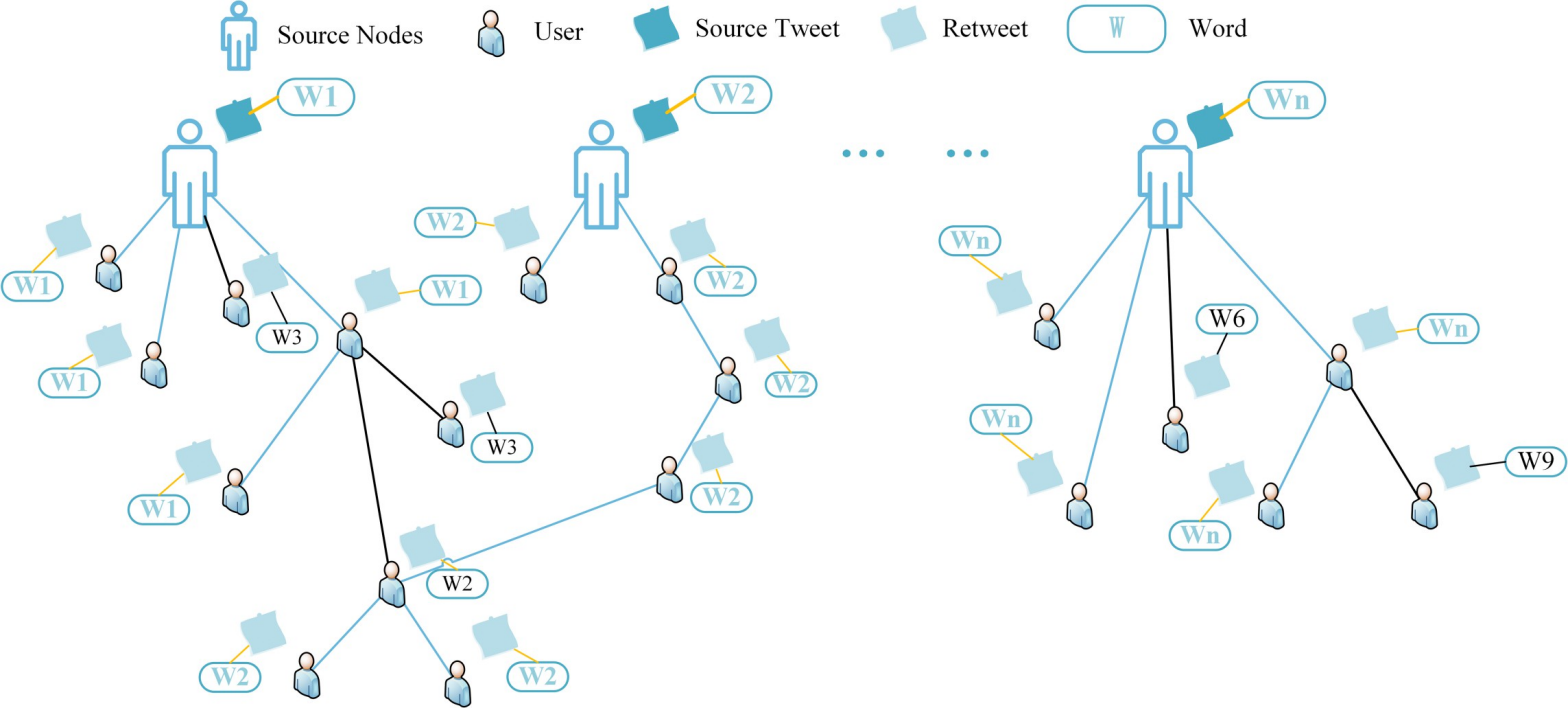
Source tweet-word propagation graph of *S*_*ti*_.

This paper considers the small-world properties of social networks and constructs a Twitter global propagation graph based on the rumor propagation structure, as shown in Fig 1. In the present paper, The Twitter global graph *G* = (*V*,*E*), where V and E represent nodes and edges in the graph. Node V represents the source tweet or retweet corresponding to the user node and the words it contains, which is constructed based on the propagation process of all tweets in the dataset. This study defines each participating user in the propagation as a node, and the global graph includes all the nodes in the propagation process of the source tweet. However, each node in the Twitter global graph has different importance for learning node embeddings for rumor detection and suffers from data sparseness in the early stage of information propagation. To more accurately learn the semantic association representation between the source tweet text and the retweeted text, the source tweet-word propagation graph can be obtained by decomposing the global graph. The propagation graph of different source tweet *S*_*ti*_ is shown in Fig 2.

Specifically, this study defines each user who participates in the dissemination of source tweets as a node, the user who sends out the source tweet is defined as a source node, and each user node contains the tweet text and related words corresponding to the user. In Fig 2, the orange edge represents the co-occurrence word in the retweet corresponding to the source tweet. The black edge represents the edge with no opinion association between nodes in the propagation process, and the blue edge indicates that the node has opinion correlation or co-occurring words in the corresponding tweet during the propagation process.

In the decomposed source tweet-word graph, there are two forms of edge *E*: Connected edges between source tweets and words *E*_*sw*_, Connected edges between tweets corresponding to node *V* with opinion similarity *E*_*sr*_. *E*_*sw*_ denotes the relationship of the source tweet to the words it contains. *E*_*sr*_ denotes indicates that the tweets of different users have opinion similarity or their tweets contain co-occurring words.

### 3.2 Problem statement

In this study, given a constructed source tweet-word graph *G* = (*V*,*E*), Where *V* = {*S*, *O*}, *E* = {*E*_*sw*_, *E*_*sr*_} denote nodes and edges in the global graph, respectively. *T* represents all tweets corresponding to nodes *V*, *S*_*ti*_ denotes the collection of the i-th source tweet and its retweets, i.e. *T* = {*S*_*t*1_, *S*_*t*2_,…,*S*_*tn*_}, where n is the number of source tweets. *W* denotes the set of words contained in the tweet, i.e. *W* = {*w*_1_, *w*_2_,…,*w*_*m*_}, where m is the total number of words in the set of words. *O* denotes the set of tweets with opinion similarity corresponding to nodes *V*. *E*_*sw*_, *E*_*sr*_ represents the edge of the source tweet and the words it contains, the edge with co-occurring words or opinion similarity between the source tweet and the retweet, respectively.

Generally speaking, rumor detection is transformed into a binary classification task to determine whether news or information circulating on social media is a rumor. A classifier can be formalized as a function that determines whether *y* is a rumor or not. In this paper, for obtaining an effective classifier, the proposed model will learn the function *p*(*c*|*S*,*G*,*θ*) to determine the label probability of the set of tweets *S*_*ti*_. *c* and *θ* represent the class labels and model parameters to be learned, respectively, and the studied model is constructed based on the graph attention network.

### 3.3 The overall framework of the proposed model STWA

**Fig 3:**
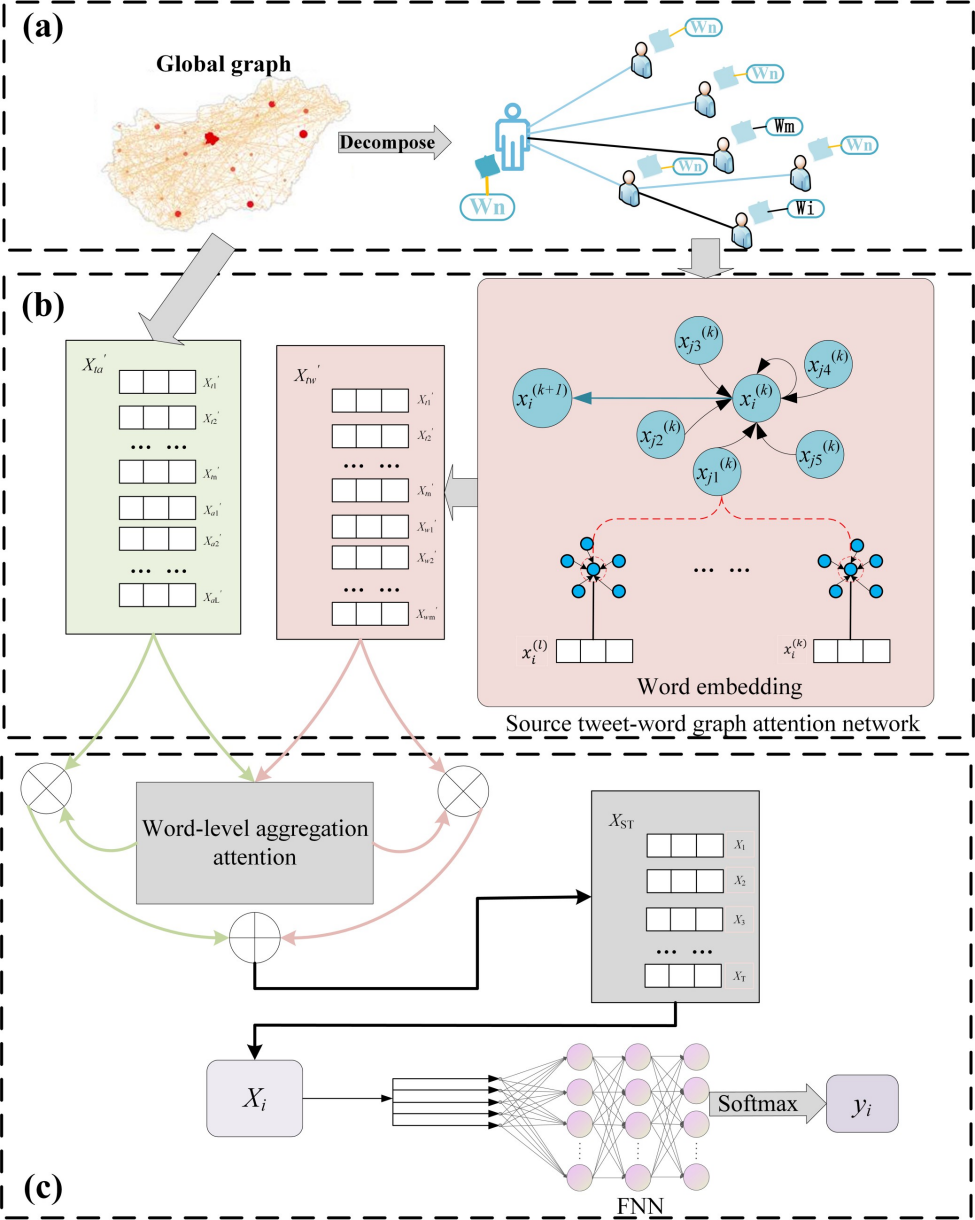
Overview of STWA: (a) input of STWA; (b) processing layer; (c) text semantic association learning and rumor detection layer.

In this subsection, the overall framework of the proposed method STWA is described. As shown in Fig 3, it contains (a) the input layer: which includes (1) a global spread graph of all tweets in the dataset. (2) *S*_*ti*_’s Source Tweet-Word Graph. (b) Processing layer, which includes (1) the source tweet-word graph attention network, which utilizes the attention mechanism of graph attention network [29] to capture the semantic association representation of source tweet text content and retweet text content in global propagation. (2) Text Attention Mechanism Based on Opinion Similarity, which uses attention mechanism to fuse twitter text content representations with opinion similarity [30] in the process of different source tweet propagation for rumor detection. (c) Text semantic association representation learning layers and rumor detection layers. The above will be explained in detail next.

The STWA will make full use of the source tweet text content to learn the contextual semantic association representation in the small-world network propagation process. Afterward, the text attention mechanism based on opinion similarity is used to fuse all textual content and contextual semantic association representations in different source tweets to achieve the purpose of early rumor detection.

### 3.4 Source tweet-word graph attention network

To capture the semantic association between source tweets and retweets. This paper considers the small-world property in Twitter networks and is inspired by graph attention networks. The multi-head attention mechanism in the graph attention network is used to model and analyze the source node and its neighbor nodes with a large aggregation coefficient, and give a higher weight to the neighbor nodes that have a shorter propagation path than the source node. Word embeddings are then generated through a graph neural network to learn semantic association representations in the context of the source tweet.

Therefore, in this study, the source tweet-word graph is modeled based on the decomposition of global graph. Construct edge *E*_*sw*_ of the source tweet and the words it contains, and the edge *E*_*sr*_ with co-occurring words or opinion similarity between the source tweet and the retweet. The weight of the edge *E*_*sw*_ can be obtained by computing the term frequency-inverse document frequency (TF-IDF) [31] of the words in the source tweet. The weights that define edge between node *i* and node *j* are calculated as follows:

$$W_{ij}=\left\{ \begin{array}{l} {TF-IDF}_{ij},\;i\;is\;source\;tweet,\;j\;is\;word\\ PMI(i,j),\;i\;is\;source\;tweet,\;j\;is\;retweet\\ \frac{1}{t_{ij}+1},\;i\;is\;retweet,\;j\;is\;word\\ 1,\;i=j\\ 0,\;Otherwise \end{array}\right.$$ (1)

Where *t* denotes the elapsed time for the retweet *i* related to the word *j*. Therefore, the length of elapsed forwarding time can be used to judge the connection strength between node *i* and node *j* in the small-world network. The *TF*−*IDF* values of source tweet *i* and word *j* are calculated as follows [31]:

$${IDF}_{j}=\log\frac{\left|\tau \right|}{\left|\left\{k:\omega_{j}\in t_{k}\right\}\right|}$$ (2)

Where |*τ*| represents the total number of tweets. |{*k*: *ω*_*j*_∈*t*_*k*_}| denotes the number of tweets that contain word *j*. The *PMI* value [32] of the word corresponding to source tweet *i* and retweets *j* in *PMI*(*i*,*j*) is calculated as:

$$PMI(i,j)=\log\frac{p(i,j)}{p(i)p(j)}$$ (3)

Where *p*(*i*) and *p*(*j*) can be calculated by referring to work [31].

In the spread graph of the source tweet *S*_*ti*_, the tweet word *W* corresponding to each node is defined as $X_{W}=\{x_{w1},x_{w2},\ldots ,x_{wm},\},x_{wi}\epsilon R^{N}$, *x*_*wi*_ is the word embedding representation of word *w*_*i*_. The *T* is denoted as $X_{T}=\{x_{t1},x_{t2},\ldots ,x_{tn},\},x_{ti}\epsilon R^{N}$, where the calculation formula of embeddings *x*_*ti*_ is the average value of the word representations contained in its corresponding tweet *t*_*i*_. In particular, the *x*_*t*1_ representation is computed from the source tweet *S*_*t*1_. Its calculation formula is [31]:

$$x_{ti}=\frac{1}{\left|t_{i}\right|}\sum_{w_{i}\in t_{i}}x_{w_{i}}$$ (4)

Next, define the nodes *V* in the source tweet-word propagation graph are denoted as $X_{tw}=\{{x_{t1},x_{t2},\ldots ,x_{tn},x}_{w1},x_{w2},\ldots ,x_{wm}\},\;{x_{ti}\epsilon X_{T},\;x}_{wi}\epsilon X_{W}$. A self-attention propagation graph is then used to learn weights between nodes. The calculation formula of the attention coefficient *e*_*i*,*j*_ of a node pair (*i*,*j*) in a given propagation graph is as follows [33]:

$$e_{i,j}=f(\omega_{x_{i}},\omega_{x_{j}}),x_{i},x_{j}\in X_{tw}$$ (5)

Then, the attention is randomly masked and the structural information of source tweet-word graph is introduced into the model. Normalize them with the softmax function to obtain the coefficients *α*_*i*,*j*_ [31]:

$$\alpha_{i,j}=softmax(e_{i,j})=\frac{exp(\sigma (a^{T}∙[\omega_{x_{i}}\|\omega_{x_{j}}]))}{\sum_{k\epsilon N_{i}}exp(\sigma (a^{T}∙[\omega_{x_{i}}\|\omega_{x_{k}}]))}$$ (6)

Then aggregate the neighbor representations of node *i* and their corresponding coefficients in the propagation graph to update the embedded representation of node *i* and perform K transformations. The final output representation is as follows:

$$x_{i}^{(1)}=\sigma \left(\sum_{j\in N_{i}}\alpha_{i,j}\omega_{x_{j}}\right)$$

$$x_{i}^{\prime }={\left|\right|}_{k=1}^{K}\sigma \left(\sum_{j\in N_{i}}\alpha_{i,j}^{k}{\omega^{k}}_{x_{j}}\right)$$ (7)

Where $\alpha_{i,j}^{k}$ represents the normalized attention coefficient obtained by the kth attention mechanism (*f*^*k*^), *ω*^*k*^ Represents the weight matrix corresponding to the input linear transformation [31].

Define the representation *X*_*tw*_ of node *V* in the source tweet-word propagation graph, after feeding the node representation into the propagation graph attention network, the node embedding $X_{tw}^{\prime }=\{x_{t1}^{\prime },x_{t2}^{\prime },\ldots ,x_{tn}^{\prime },x_{w1}^{\prime },x_{w2}^{\prime }\ldots ,x_{wm}^{\prime }\}$ can be obtained using the source tweet-word graph with global semantic association information.

### 3.5 Text attention mechanism based on opinion similarity

In addition to obtaining the contextual semantic relationship between source tweets and retweets in the source tweet-word graph. In order to learn text semantic association representation for tweets corresponding to user nodes with weak link strength in the rumor dataset, this study applies a word-level aggregated attention mechanism in the processing layer.

The network in the early stage of information dissemination is often sparse. Therefore, it can be considered to increase the embedding representation in the global graph for more accurate node embedding learning. In this study, in order to determine whether the tweet opinions between the retweets and the corresponding user nodes of the source tweet are similar. Opinion similarity is introduced to determine the weight of edge *E*_*sr*_ in global graph, which can help to obtain the opinions to further learn contextual semantic association representations between tweets.

Therefore, a word attention network is established by obtaining node embeddings based on opinion features, and the edge *E*_*sr*_ weight is calculated by formula (1). The *PMI*(*i*,*j*) value is calculated as follows:

$$PMI(i,\;j)=\log\frac{p(i,j)}{p(i)p(j)}$$

$$p(i)=\frac{O_{i}}{\#O}$$ (8)

$$p(i,j)=O_{\left(i,j\right)}$$

Where *O*_*i*_ represents the number of opinion words containing node *i*, #*O* represents the total number of tweet words, and *O*_(*i*,*j*)_ represents the opinion similarity probability between node *i* and node *j*.

In the global graph-based case, same as section 3.3, define the representation *X*_*ta*_ of the word node *V* in the Twitter global propagation graph, the node embedding $X_{ta}^{\prime }=\{x_{t1}^{\prime },x_{t2}^{\prime },\ldots ,x_{tn}^{\prime },x_{a1}^{\prime },x_{a2}^{\prime }\ldots ,x_{aL}^{\prime }\}$ is obtained by passing the word representation of the nodes in the global graph through a single-layer graph convolutional neural network.

Afterwards, the graph attention mechanism is used to fuse all tweet text content representations in the process of tweet propagation from different sources to further learn the word node weights for rumor detection and calculate the importance of different node embeddings. Taking the node embeddings $X_{tw}^{\prime }$ and $X_{ta}^{\prime }$ as input, the weights of the source tweet-word graph and the global graph are calculated as follows [34]:

$$\left(\beta_{tw},\beta_{ta})={att}_{gra}(X_{tw}^{\prime },X_{ta}^{\prime }\right)$$

$$\omega_{tw(ta)}=\frac{1}{\left|X_{tw(ta)}^{\prime }\right|}\sum_{x_{i}\epsilon X_{tw(ta)}^{\prime }}a^{T}∙\tanh\left(\omega_{gra}x_{i}\right)$$ (9)

$$\beta_{tw(ta)}=\frac{exp(\omega_{tw(ta)})}{\sum_{\Phi \in \{tw,ta\}}exp(\omega_{\Phi })}$$

Finally, using the learned propagation graph weight coefficients and fusing the representations of tweet nodes in the two propagation graphs, the representation *X*_*ST*_ of the source tweet is obtained as follows

$$X_{ST}=\{x_{1},x_{2},\ldots ,x_{T}\}$$

$$x_{i}=\sum_{\Phi \in tw,ta}\beta_{\Phi }∙x_{t_{i}},x_{t_{i}}\in X_{\Phi }^{\prime }$$ (10)

Where $x_{t_{i}}$ represents the representation of a tweet node *i* with global textual association information in the propagation graph Φ. $X_{\Phi }^{\prime }$ represents all node representations in the propagation graph Φ with global textual association information.

### 3.6 Output layer

In the rumor detection layer of the STWA model, this study combines the source tweet-word graph attention mechanism with the output vectors of the opinion similarity-based text aggregation attention mechanism. The output dependent variable is to compute the rumor label *y*_*i*_ of the tweets to predict the class probability distribution of the source tweet:

$$p(c|S_{ti},G;\theta )=softmax(FNN(x_{i})),x_{i}\epsilon X_{ST}$$ (11)

The function is formalized as follows:

$$L=-\sum_{i\in |T|}y_{i}p(c|S_{ti},G;\theta )+\lambda |{\left|\theta |\right|}_{2}^{2}$$ (12)

Where *y*_*i*_ represents the one−hot encoding of the ground truth of the *i*th source tweet. Using L2 regularization to prevent the occurrence of overfitting.

The model acquires more tweet text content information and semantic association representations of source tweet text and retweet text in two modules, which make use of almost all the text information in the rumor dataset.

The adaptive learning rate optimization algorithm Adam [35] is used for model training. Detailed aspects of the computational experiments are provided in the section 4.

## 4. Experiments and results

In this section, this study experimentally evaluates the model performance of STWA, and compares with existing baselines to verify the performance of the proposed model on rumor detection and early rumor detection tasks.

### 4.1 Experimental data

In this study, the performance of the model is validated on two publicly available real-world Twitter datasets. Ma et al. [36] collected these data in previous research work and named Twitter15 and Twitter16. They contain 739 and 404 source tweets, respectively (For detailed data see Table 1). Each source tweet in the dataset is labeled as non-rumor (NR), false rumor (FR), true rumor (TR), or unverified rumor (UR) [37].

**Table 1:** Statistics of the datasets.

Statistic	Twitter15	Twitter16
# Source tweets	739	404
# Users	306,402	168,659
# Tweets	331,612	204,820
Max. # retweets	2,990	999
Min. # retweets	97	100
Avg. # retweets	493	479
Avg. # time length	743h	167h
# Non-rumors	374	205
# False-rumors	370	205
# True-rumors	372	207
# Unverified rumors	374	201

### 4.2 Parameter settings

In this study, referring to the experimental parameter settings in works [21, 38], 10% of the data set was randomly selected as the validation set of the experiment, and the training set and test set were set at a ratio of 3:1 during model training.

The proposed method STWA is implemented by PyTorch in Python 3.8. During the training process, the performance of the model is finally verified on the test set. For the setting of model parameters, the attention network parameter K of the propagation graph is recommended to be set to 8 and the training batch size to 128.

### 4.3 Baselines

The rumor detection method proposed in this paper will be compared with the following baseline experiments:

- DTC: A method for collecting statistical features of tweets, using decision trees to extract tweet features [10].
- RFC: A manual feature extractor that fits temporal attributes to parameters corresponding to user, content, and structural features [11].
- SVM-TK: A SVM Classifier for Calculating Rumor Similarity Using Propagation Tree Structure [36].
- GRU-RNN: An RNN with gated recurrent units to capture time-series information capable of learning the sequential structure of tweets for rumor detection [5].
- BU-RvNN and TD-RvNN: An RNN based on propagation trees that can obtain propagation features and context semantics [19].
- PPC: A method for capturing propagation paths that fuse recurrent and convolutional networks [21].
- GCAN: A co-attention network-based approach to detect rumors by fusing Source tweet text content and users’ information [27].
- Rumor2vec: A method that learns the correlation representation between text content and communication structure by constructing a communication graph based on the Twitter communication structure [6].

Since the micro-average precision (i.e. Acc.) is a measure of whether the test set is a rumor and the classification is correct. the F1 score is the harmonic mean of precision and recall. Therefore, for a fair comparison with the baselines, in this study, the micro-average accuracy Acc. and F1 score can be used to evaluate the proposed method STWA.

### 4.4 Rumor detection

The experimental results can be significantly observed in Tables 2 and 3, where NR denotes non-rumors, FR denotes false rumors, TR denotes true rumors, UR denotes unconfirmed rumors, and the bold value represents the highest value in the category. The experimental results show that the overall performance of STWA on Twitter15 and Twitter16 datasets outperforms all baseline models.

**Table 2:** Overall performance comparison of rumor detection on Twitter15.

Method	Accuracy	F1(NR)	F1(FR)	F1(TR)	F1(UR)
DTC	0.454	0.733	0.355	0.317	0.415
SVM-TK	0.667	0.619	0.669	0.772	0.645
GRU-RNN	0.641	0.684	0.634	0.688	0.571
BU-RvNN	0.708	0.695	0.728	0.759	0.653
TD-RvNN	0.723	0.682	0.758	0.821	0.654
PPC	0.842 ^a^	0.818	0.875	0.811	0.790
Rumor2vec	0.796	0.883	0.746	0.836	0.723
**STWA**	**0.911** ^b^	**0.935**	**0.912**	**0.922**	**0.874**

^a^The second best one.

^b^The best method.

**Table 3:** Overall performance comparison of rumor detection on Twitter16.

Method	Accuracy	F1(NR)	F1(FR)	F1(TR)	F1(UR)
DTC	0.465	0.643	0.393	0.419	0.403
SVM-TK	0.662	0.643	0.623	0.783	0.655
GRU-RNN	0.633	0.617	0.715	0.577	0.527
BU-RvNN	0.718	0.723	0.712	0.779	0.659
TD-RvNN	0.737	0.662	0.743	0.835	0.708
PPC	0.863 ^a^	0.843	0.898	0.820	0.837
Rumor2vec	0.852	0.857	0.769	0.927	0.850
**STWA**	**0.937** ^b^	**0.917**	**0.909**	**0.952**	**0.921**

^a^The second best one.

^b^The best method.

Further observations show that traditional machine learning-based methods (DTC, SVM-TK) perform poorly, mainly because they use features based on hand-crafted statistics of tweets, and both methods are insufficient to capture the propagation structure features related to tweet text. Notably, SVM-TK outperforms DTC mainly because it exploits additional temporal or structural features in the feature set.

As for deep learning-based methods (BU-RvNN, TD-RvNN, PPC, and Rumor2vec), they have better performance than machine learning-based methods. The results in BU-RvNN, and TD-RvNN show that it is effective to study and model the propagation structure and temporal information of rumors. The results of PPC show that both user features and text features are important for rumor detection, and Rumor2vec, which is better than other baselines, shows that the method of jointly learning alliance graph and text content representation has achieved good results. The proposed method STWA outperforms all other baselines on datasets. Compared with the sub-optimal baseline models PPC and Rumor2vec, SWTA learns rumor representations only from textual content without requiring any user-profiles, proving the main motivation of this work—Semantic association between contextual texts in rumor propagation plays an important role in the early detection of rumors.

### 4.5 Early rumor detection

**Fig 4:**
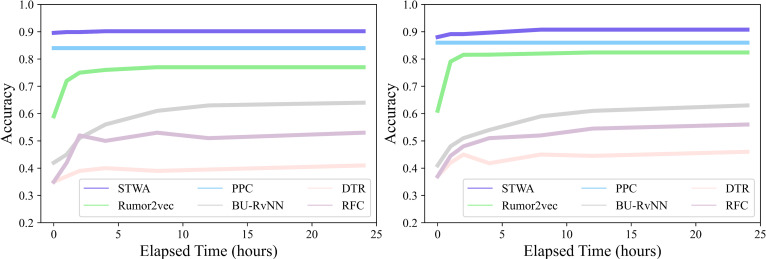
Results of rumor early detection (Elapsed time). (a) Early 24 hours (Twitter15). (b) Early 24 hours (Twitter16).

**Fig 5:**
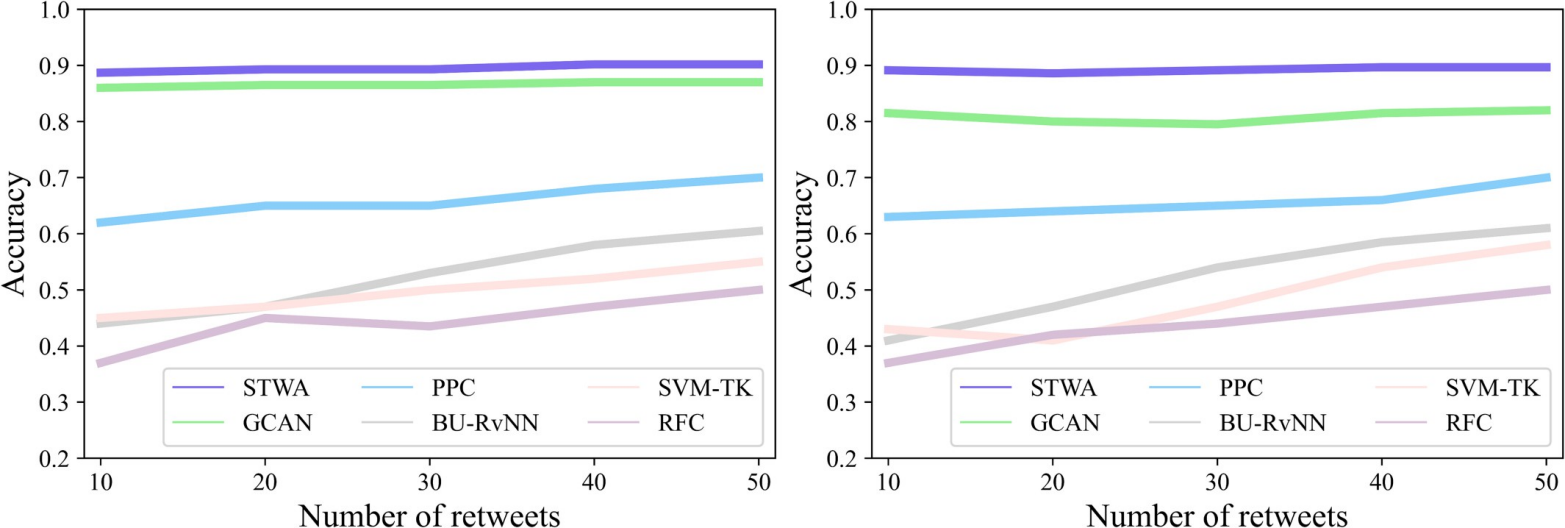
Results of rumor early detection (Number of retweets). (a) Early 50 retweets (Twitter15). (b) Early 50 retweets (Twitter16).

Early detection of rumors has always been one of the most difficult problems in this field. The original intention of STWA is to detect rumors at an early stage of their propagation and improve the accuracy of early detection. To achieve the task, this paper refers to work [4] and work [6], respectively constructing a data set of rumors in the early stage by the elapsed time and the number of retweets after the source tweet was published, and the performance of STWA on early detection task is evaluated by the detection accuracy curve. As shown in Figs 4 and 5, the elapsed time after the source tweet is published is defined as the time when the source tweet appears on social media, and the set detection points are 0, 1, 2, 4, 8, 12, and 24 hours and the number of retweets is set to 10, 20, 30, 40 and 50 respectively. On the early detection task, this paper will evaluate the early rumor detection performance of STWA based on the elapsed time and the number of retweets, respectively, and compare it with several baselines, namely DTR, RFC, BU-RvNN, PPC, Rumor2vec, and GCAN.

The experimental results are shown in Figs 4 and 5. As can be seen from the figures, whether based on elapsed time or the number of retweets, STWA has consistently very good performance on the early detection task and outperforms other baseline methods.

It can be observed from Fig 4 that when t = 0, the method proposed in this paper has reached a very high accuracy rate on Twitter15 and Twitter16. With the increase of time, the accuracy of the model also has a small improvement and remained stable. This suggests that STWA can obtain more information from the textual content embedded in the source tweet. As time increases, the proposed method acquires more information about the propagation structure and the textual content of retweets. As can be seen, its performance improves over time.

From Fig 5, it can be observed that under the limit of 50 retweets in the early stage, although the performance of GCAN on Twitter15 is close to that of STWA, its performance on Twitter16 degrades significantly. The reason is that the data volume of Teitter16 is almost half of that of Twitter15, and the sparsity of the data in Twitter16 makes some models that need to learn the representation of text content unable to achieve good performance. Compared with baselines, STWA still has better robustness and stable performance in the case of sparse data, which benefits from STWA’s effective learning of contextual semantic association representations.

### 4.6 Importance analysis of source tweet-word graph attention networks

In this subsection, to evaluate the importance of the source tweet-word graph attention network for the STWA model, we conduct ablation experiments to verify the rumor detection performance of the model in the absence of the source tweet-word graph attention network. Learning text content representations in global graph for rumor detection using only a model with global graph attention mechanism in the validation context. The experimental results are shown in scatter plot 6, w/o STWA represents a model that removes the source tweet-word graph attention network.

**Fig 6:**
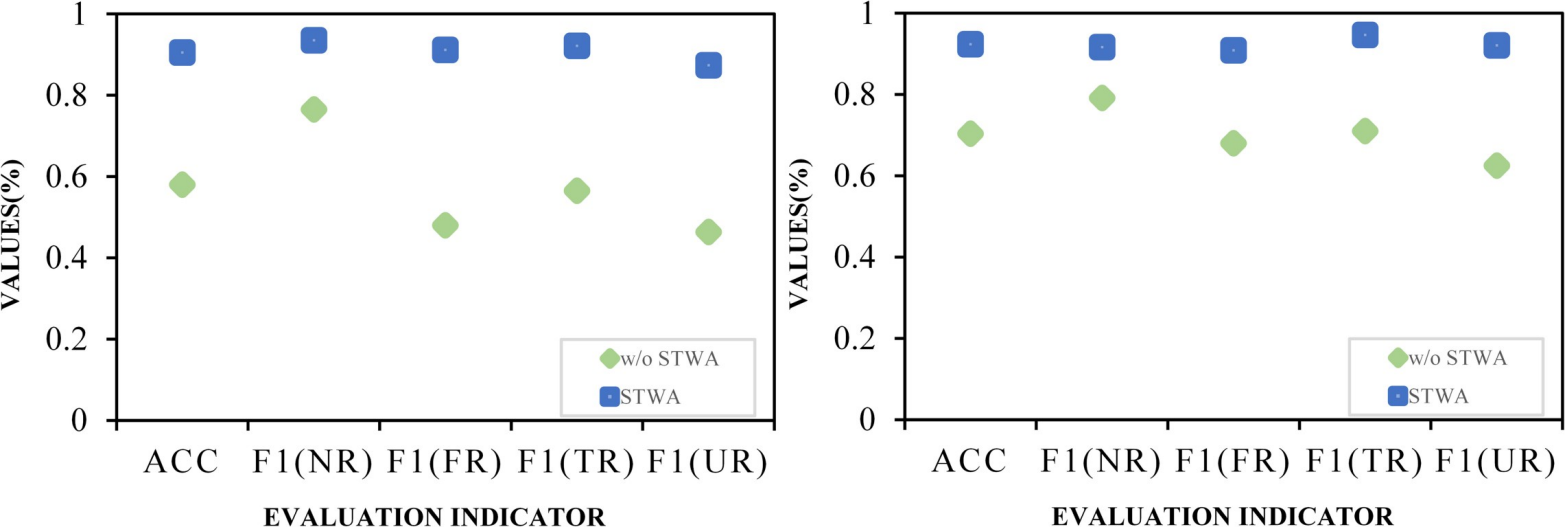
The importance analysis of Source tweet-word graph attention networks. (a) Ablation study (On Twitter15). (b) Ablation study (On Twitter16).

From the experimental results in Fig 6, it can be observed that the source tweet-word graph decomposed by the global propagation graph has a significant impact on the STWA detection framework. Specifically, it can be seen from the figure that when the source tweet-word graph attention mechanism is not added to the model, the detection accuracy of the model drops by 32.5% and 22% on the datasets. This result shows that obtaining the propagation structure of source tweets is indispensable for improving the accuracy of rumor detection of STWA. It also illustrates that learning the contextual semantic association representation between source tweets and retweets is very important for the improvement of rumor detection accuracy.

## 5. Conclusion

User nodes in the Twitter network have small-world properties with large aggregation coefficients and short propagation paths. To learn more features from the source tweet text and its propagation structure to achieve early and accurate detection of rumors. This paper constructs a global graph based on source tweet propagation structure and the decomposed source tweet-word graph and proposes a novel method STWA, which is a rumor detection method based on the graph attention network mechanism to capture as much as possible the global semantic relational representation of the tweet text content. Compared with previous rumor detection work based on text content and propagation structure, the method proposed in this paper focuses more on the early data-sparse problem of information dissemination and the learning of the semantic association representations between the source tweet text and the retweet text during the propagation process. The model can learn as many explicit and implicit representations of tweet text content as possible.

Experimental results on two public Twitter social network datasets show that the proposed rumor detection framework STWA has better rumor detection performance than existing baselines, especially in early rumor detection tasks. The method in this paper still has good robustness and stable performance in the case of sparse data.

In future work, on the one hand, the user profile information in the social network can contribute to the analysis of user node confidence. The user profiles in the dataset can be added to the model to further improve performance. On the other hand, it can be considered to achieve multimodal rumor detection tasks through semantic feature extraction of videos or pictures.

## Supporting information

S1 File The minimal data set. (ZIP)
